# Heritability of behavioural tolerance to high CO_2_ in a coral reef fish is masked by nonadaptive phenotypic plasticity

**DOI:** 10.1111/eva.12483

**Published:** 2017-05-24

**Authors:** Megan J. Welch, Philip L. Munday

**Affiliations:** ^1^ ARC Centre of Excellence for Coral Reef Studies James Cook University Townsville QLD Australia; ^2^ College of Marine and Environmental Sciences James Cook University Townsville QLD Australia

**Keywords:** behaviour, genetic variation, ocean acidification, parent–offspring regression, phenotypic plasticity

## Abstract

Previous studies have demonstrated limited potential for acclimation of adversely affected olfactory behaviours in reef fishes under elevated CO_2_, indicating that genetic adaptation will be required to maintain behavioural performance in the future. Adaptation depends on the presence of heritable phenotypic variation in the trait, which may differ between populations and environments. We used parent–offspring regressions to estimate the heritability (*h* ^2^) of variation in behavioural tolerance to high CO_2_ (754 μatm) in both field‐collected and laboratory‐reared families of *Acanthochromis polyacanthus*. Tolerance to elevated CO_2_ was measured by determining the behavioural response of individuals to chemical alarm cues. Both populations exhibited high heritability of olfactory behaviour phenotype (father–mid‐offspring *h* ^2^ = 0.56 & 0.65, respectively) when offspring were acutely exposed to high CO_2_ for 4 days. However, there was no heritability in the behavioural phenotype when juveniles were chronically exposed to high CO_2_ for 6 weeks in the laboratory‐reared families. Parental exposure to high CO_2_ during the breeding season did not alter this relationship between heritability and length of juvenile exposure to high CO_2_. These results demonstrate that variation in behavioural tolerance to high CO_2_ is heritable, but adaptive potential may be constrained by a loss of phenotypic variation when juveniles permanently experience a high‐CO_2_ environment, as will occur with rising CO_2_ levels in the ocean.

## 1 INTRODUCTION

Ocean acidification, caused by the uptake of additional CO_2_ from the atmosphere (Caldeira & Wickett, 2003), will impact many marine species and have far‐reaching effects on the ecosystems they inhabit (Gattuso & Hansson, 2011). However, some species might be able to adapt to the projected changes in ocean chemistry, which could reduce the impacts on populations and communities (Gaylord et al., 2015; Sunday et al., 2014). Short‐term experiments have demonstrated negative effects of future ocean acidification on a wide range of marine species and ecological processes (Doney, Fabry, Feely, & Kleypas, 2009; Kroeker et al., 2013), yet few studies have attempted to assess the potential for adaptation. Nevertheless, the limited number of studies conducted to date illustrate that some species exhibit heritable phenotypic variation in response to ocean acidification (Kelly, Padilla‐Gamiño, & Hofmann, 2013; Malvezzi et al., 2015) and that selection of CO_2_‐tolerant genotypes can occur (Malvezzi et al., 2015; Pespeni et al., 2013). In some other species, however, there appears to be limited heritable variation of phenotypic traits at high CO_2_ (Sunday, Crim, Harley, & Hart, 2011), or there are genetic correlations with other environmental stressors that could limit adaptive potential (Foo, Dworjanyn, Khatkar, Poore, & Byrne, 2014). Assessing the potential for adaptation in a broader range of marine taxa and phenotypic traits affected by ocean acidification is therefore a priority.

Recent studies show that CO_2_ levels predicted for the end of the century can have adverse effects on the behaviour of marine fishes (Briffa, de la Haye, & Munday, 2012; Clements & Hunt, 2014; Heuer & Grosell, 2014) with consequences for key ecological processes such as larval dispersal, habitat selection, competition and predator–prey interactions (Nagelkerken & Munday, 2016). Many reef fishes innately recognize ecologically relevant olfactory cues, such as predator odour and chemical alarm cues (CAC) from injured conspecifics, and they use these cues to avoid danger (Dixson, Pratchett, & Munday, 2012; Holmes & McCormick, 2010). Under elevated CO_2_, however, reef fish lose the ability to appropriately interpret these cues (e.g., Chivers et al., 2014; Dixson, Munday, & Jones, 2010; Ferrari, Dixson, et al., 2011; Ferrari, Manassa, et al., 2012; Nilsson et al., 2012). Altered responses to predation threats may have significant population‐level effects due to increases in juvenile mortality, which can affect population replenishment (Chivers et al., 2014; Ferrari, Dixson, et al., 2011; Ferrari, McCormick, et al., 2011; Munday et al., 2010). Altered olfactory responses persist for weeks to months in elevated CO_2_ conditions (Munday, Cheal, Dixson, Rummer, & Fabricius, 2014; Munday et al., 2013), and transgenerational experiments further demonstrate that impaired behaviours are not ameliorated when parents are held under the same elevated CO_2_ levels as their offspring (Welch, Watson, Welsh, McCormick, & Munday, 2014). Nevertheless, previous studies have observed individual variation in olfactory tolerance to elevated CO_2_ (Welch et al., 2014), especially at near‐future CO_2_ levels around 700 μatm (Ferrari, Dixson, et al., 2011; Munday et al., 2010). Furthermore, selection for CO_2_‐tolerant behavioural phenotypes has been observed in field‐based experiments (Munday et al., 2012). This phenotypic variation and natural selection could be key for future adaptation; however, it is unknown whether variation in behavioural tolerance to high CO_2_ is heritable in coral reef fishes.

A common observation from evolutionary studies is that heritability is not constant and can vary with environmental conditions (Hoffmann & Merilä, 1999). This potentially complicates attempts to assess adaptive potential to climate change and ocean acidification because adaptation could be less likely under some environmental conditions than others. Many different hypotheses have been proposed to explain why heritability can vary among environments (reviewed by Hoffmann & Merilä, 1999); however, one hypothesis relating to assessing adaptive potential to rapid environmental change is that phenotypic variation may be either increased or decreased by environmental stress (Hoffmann & Hercus, 2000). Broad‐ and narrow‐sense heritability have been observed to decline in unfavourable conditions in a variety of animals (Charmantier & Garant, 2005; Wilson et al., 2006), suggesting that heritability may also be lower in populations exposed to chronic stress from climate change. On the other hand, heritability may also increase when a stressful environment is encountered (Hoffmann & Hercus, 2000; Hoffmann & Parsons, 1991). Heritability may be higher in a stressful (or heterogeneous) environment if increased stress causes greater expression of phenotypic variation compared with less stressful (or homogenous) environments. Therefore, estimates of heritability may differ in populations exposed to an acute environmental stress compared with chronic environmental change. The effects of acute versus chronic environmental stress are especially relevant to experiments testing adaptive potential to ocean acidification because such experiments often expose juveniles or adults to high CO_2_ for just a few days or weeks (i.e., acutely), whereas ocean acidification will result in the permanent (i.e., chronic) exposure to high CO_2_ throughout life.

An additional complication of assessing the adaptive potential to high CO_2_ is that parental exposure to environmental stress can alter offspring phenotypes independently of genetic variation (Guillaume, Munro, & Marshall, 2016). Parents can influence the phenotype of their offspring through a range of nongenetic mechanisms that involve the transmission of nutrients, hormones, somatic factors or epigenetic marks (Bonduriansky & Day, 2009). Commonly, mothers influence the phenotype of their offspring in different environments through changes in the provisioning of eggs, embryos and juveniles (maternal effects) (Crean & Marshall, 2009; Marshall, 2008; Mousseau & Fox, 1998). There is also increasing evidence that the environmental conditions experienced in one generation can influence future generations through the inheritance of different epigenetic states (epigenetic inheritance) (Bonduriansky, Crean, & Day, 2012; Holeski, Jander, & Agrawal, 2012; Jablonka & Lamb, 1995). Consequently, the environment experienced by parents can influence the expression of phenotypic variation in their offspring through a range of nongenetic mechanisms (Bonduriansky, 2012; Day & Bonduriansky, 2011). Recent studies have shown that parental exposure to high CO_2_ can ameliorate the negative effects of high CO_2_ on growth and survival of juvenile fish (Miller, Watson, Donelson, McCormick, & Munday, 2012; Murray, Malvezzi, Gobler, & Baumann, 2014) and can also influence the kinematic responses of juveniles to a perceived threat (Allan, Miller, McCormick, Domenici, & Munday, 2014). Similar beneficial parental effects have not been observed in the average response of juvenile fish to olfactory cues in a high‐CO_2_ environment (Welch et al., 2014). Nevertheless, parental effects could potentially influence the variation in behavioural responses to high CO_2_ exhibited by offspring, and thus the heritability of CO_2_ tolerance.

There are significant impediments to testing heritability of phenotypic traits in coral reef fishes because (i) the life cycle of most reef fish species cannot be completed in captivity due to difficulties rearing the small pelagic larva and (ii) those species that can be readily reared in captivity are demersal spawners with paternal egg care, making it practically impossible to cross‐fertilize eggs and sperm in a diallel breeding design (Rummer & Munday, 2016). The spiny damselfish, *Acanthochromis polyacanthus,* is a monogamous species of reef fish that broods each clutch of offspring for several months after hatching (Kavanagh, 2000). Furthermore, this species can be reared with high success in the laboratory. Thus, it is ideally suited to estimating heritability with parent–offspring regressions (Falconer & Mackay, 1996; Lynch & Walsh, 1998), which is the approach used here.

In this study, we used parent–offspring regressions to test for heritability of variation in behavioural responses to CAC under elevated CO_2_ conditions in two different populations of *A. polyacanthus*. First, we assessed the heritability of variation in behavioural responses to CAC following acute exposure to elevated CO_2_ in field‐collected families of *A. polyacanthus*. Next, we tested for heritability of the same trait following acute exposure to elevated CO_2_ in laboratory‐reared families of the same species. In the laboratory‐reared population, we were also able to test for heritability of variation in behavioural responses to CAC following chronic exposure of juveniles to elevated CO_2_ and also when parents had been chronically exposed to high CO_2_. Consequently, the laboratory‐reared population enabled us to investigate potential differences in heritability associated with acute versus chronic exposure of juvenile fish to high CO_2_ and possible nongenetic parental effects from chronic exposure of adults to high CO_2_.

## 2 METHODS

### 2.1 Study species

The spiny chromis, *A. polyacanthus,* is found on reefs throughout the Indo‐Australian region. They form long‐term monogamous pairs and lay demersal eggs in small caves in the reef (Robertson, 1973; Thresher, 1983). Pairs typically produce one to two clutches of juveniles in a breeding season (Nakazono, 1993; Pankhurst, Hilder, & Pankhurst, 1999). Egg clutches, varying in size from 100 (Robertson, 1973) to 550 eggs (Kavanagh, 2000), are laid in a single event and cared for by both parents (Nakazono, 1993; Robertson, 1973; Thresher, 1983). Eggs hatch into small, well‐developed juveniles that remain with the parents for several months after hatching (Kavanagh, 2000).

### 2.2 Experimental approach

#### 2.2.1 Acute exposure to high CO_2_

We first tested the heritability of variation in behavioural responses to CAC following acute exposure to high‐CO_2_ conditions in field‐collected and laboratory‐reared families of *A. polyacanthus*. In both populations, parents were exposed to elevated CO_2_ (~750 μatm) for 5–7 days, while their offspring were exposed to ambient control (~450 μatm) or elevated CO_2_ for 4 days, before the behavioural response to CAC was measured. This exposure time for the acute treatment was chosen because previous studies have demonstrated that 4–5 days is sufficient time to induce the full range of behavioural effects of high CO_2_ in reef fishes (Munday et al., 2010, 2013). The high‐CO_2_ treatment level of 750 μatm was chosen to match end‐of‐century CO_2_ projections under the moderate RCP6 emissions trajectory (IPCC 2014), and because this is the CO_2_ level at which the greatest individual variation in behavioural responses to high CO_2_ has been observed in previous studies (Ferrari, Dixson, et al., 2011; Munday et al., 2010, 2012). By testing both parents and their offspring at 750 μatm CO_2_, we were able to observe the greatest phenotypic variation in behavioural response to CAC that could be used to estimate heritability of this trait. We also measured the behavioural response to CAC of sibling juveniles that were not exposed to high CO_2_, to provide a baseline with which to compare the behavioural response of juveniles to CAC following exposure to high CO_2_. Heritability in behavioural response to CAC was not estimated in ambient control conditions because adult response to CAC was only measured following high‐CO_2_ exposure, and not when adults were only in control conditions.

Juveniles were approximately 5 weeks old in the field‐collected families and were 6 weeks old in the laboratory‐reared families, which enabled us to compare the estimates of heritability in behavioural responses to CAC following acute high‐CO_2_ exposure between the two populations. Further details of the experimental procedures for both the field‐caught and laboratory‐reared populations are provided below.

#### 2.2.2 Chronic exposure of offspring to high CO_2_

To test whether chronic exposure of juveniles to high CO_2_ affects the heritability of the behavioural response to CAC, we reared a subset of sibling fish from each clutch in the laboratory‐reared population for 6 weeks at 750 μatm CO_2_ before testing their behavioural response to CAC. Sibling juveniles in the acute and chronic high‐CO_2_ treatments were tested at the same age (6 weeks) to facilitate a direct comparison between the two treatments.

#### 2.2.3 Nongenetic parental effects

**Figure 1:**
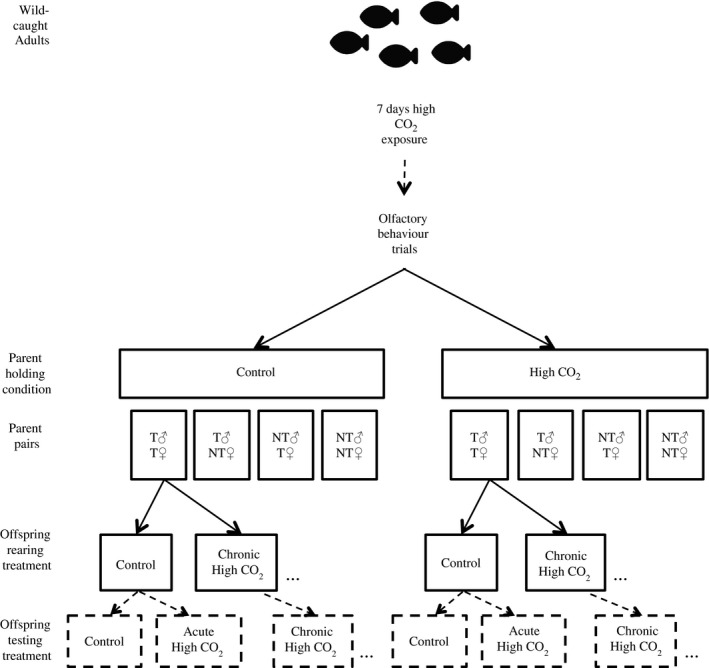
Experimental design for the laboratory‐reared population. All adults were first held in high CO_2_ for 7 days before measuring their response to chemical alarm cues. Parent pairs were constructed based on the response of individuals to chemical alarm cues, where T = tolerant and NT = nontolerant. Pair formations, as seen in “Parent pairs” boxes, were maintained under their respective treatments, control (414 μatm) and high CO_2_ (754 μatm), for breeding. Clutches of offspring from parent pairs were divided between control and high CO_2_ conditions at hatching and reared in both conditions for 6 weeks. Acute high CO_2_ refers to offspring reared in control conditions that were transferred to high CO_2_ (754 μatm) 4 days prior to behaviour testing at the end of the 6‐week rearing period

Previous studies have shown that parental exposure to high CO_2_ can affect the phenotypic response of offspring to a high‐CO_2_ environment in some traits (Miller et al., 2012), but not others (Welch et al., 2014). To test whether parental exposure to high CO_2_ altered the heritability of variation in behavioural responses to CAC, we repeated the acute and chronic high‐CO_2_ treatments described above for the laboratory‐reared families, except parents were maintained in high CO_2_ for 3 months until breeding, after their initial assessment of behavioural response to acute exposure to high CO_2_ (Figure 1).

### 2.3 Experimental procedure

#### 2.3.1 Field‐collected population

Twenty adult pairs of *A. polyacanthus* that were brooding offspring were collected from the Lizard Island lagoon on the northern Great Barrier Reef (14°40′S, 145°28′E) during November 2014. Both parents were first collected by placing a barrier net around the nest site, and the offspring were then collected using hand nets. Families were brought into the laboratory at Lizard Island Research Station (LIRS) where they were housed in 32‐L (380 L × 280 W × 300 H mm) aquaria. Parents were separated from their offspring and placed in the high‐CO_2_ treatment (754 μatm) for 5 days. Each clutch of offspring was divided equally so that half were placed in ambient control conditions (452 μatm) and half in high‐CO_2_ treatment (754 μatm) for 4 days. Temperatures and photoperiod were maintained at summer conditions (27.5°C; 13L:11D). Adult breeding pairs were fed 0.15 g of commercial fish feed pellet (INVE Aquaculture Nutrition NRD 12/20) twice a day. Juvenile groups were fed 0.05 g of commercial fish feed pellet (INVE Aquaculture Nutrition NRD 5/8) twice a day. Fish were not fed on the morning of their behaviour tests.

After the 4‐ to 5‐day treatment period, all fish were tested for behavioural responses to CAC in a two‐channel flume, as described below. Olfactory response to CAC in high CO_2_ was tested for both parents in the 20 breeding pairs. The sex of each parent was determined after behavioural testing by examining the shape of the genital papillae. Behaviour was tested for 20 offspring (10 in control and 10 in high CO_2_) from each of these breeding pairs, for a total of 400 wild‐caught juveniles. Families were returned to their collection sites after testing, except for two juveniles from each family that were retained to estimate age by examination of daily increments in the otoliths (ear bones). Sagitta were extracted and prepared following standard technique (Fowler, 1990). The average age of juveniles in the field‐caught families was 35 ± 6.7 (*SD*) days.

#### 2.3.2 Laboratory‐reared population

Adult *A. polyacanthus* were collected from the northern Great Barrier Reef, Australia, and transported to the experimental aquarium facility at James Cook University. Adults were held under high CO_2_ (754 μatm) for 7 days, after which they were tested for their response to CAC in a two‐channel flume (Figure 1). Adult sensitivity to high CO_2_ was categorized by their response in the flume. Individuals that spent ≤30% time in the cue were considered to be “tolerant” to high CO_2_, whereas individuals that spent ≥50% time in the cue were considered “nontolerant.” Adults were further categorized by size and sex. Breeding pairs were then constructed by pairing individuals of either similar or different behavioural sensitivity: tolerant male + tolerant female, tolerant male + nontolerant female, nontolerant male + tolerant female, nontolerant male + nontolerant female (Figure 1). This pairing was designed to provide the greatest possible range in parental sensitivities to high CO_2_. Assortative pairing can inflate estimates of heritability compared with random pairing (Falconer & Mackay, 1996); however, our aim was to generate a maximum possible estimate of heritability (Hill, 1970; Reeve, 1961). Adult pairs were then held in 40‐L aquaria at ambient control (414 μatm) or high CO_2_ (754 μatm) conditions for 3 months prior to the start of the breeding season. Temperatures and laboratory photoperiod were slowly adjusted during the acclimatization period to reach summer conditions (28.5°C; 13L:11D) at the start of the breeding season. Pairs were checked daily for the presence of egg clutches once the breeding season commenced. On hatching, clutches were immediately divided and transferred to control and high‐CO_2_ conditions (Figure 1). Offspring were reared under the two treatments for 6 weeks, at which point they underwent the same olfactory behavioural testing as their parents. Some offspring from control were moved to high CO_2_ (754 μatm) 4 days before the 6‐week testing to create an acute high‐CO_2_ treatment group. This acute group allowed for direct comparison with the chronic CO_2_ treatment in the laboratory‐reared population and with the acute high‐CO_2_ treatment in field‐caught population. The factorial breeding design in the laboratory further allowed for examination of any nongenetic effects of parental conditioning to elevated CO_2_ on the heritability of behavioural responses to CAC in offspring exposed both acutely and chronically to high‐CO_2_ conditions.

A total of 60 breeding pairs were formed for the laboratory experiment, 30 of which were held in control conditions and 30 in high CO_2_. Twenty pairs bred in control and 18 pairs bred under high CO_2_. Only fish from the first clutch from each breeding pair were used in the experiment. Behavioural response to CAC was tested in 60 sibling juveniles from each clutch (20 reared in control, 20 in acute high CO_2_ and 20 in chronic high CO_2_, where possible), for a total of 2,258 laboratory‐bred juveniles (752 in control, 755 in acute high CO_2_ and 751 in chronic high CO_2_).

### 2.4 CO_2_ manipulation

#### 2.4.1 Field‐collected population

Three header tanks (60 L) fed water into a total of 30 replicate 32‐L aquaria where fish were held (*N* = 10 tanks per system). One header tank was diffused with ambient air, while the other two header tanks were dosed with 100% CO_2_ to achieve the desired pH for the high‐CO_2_ treatment (754 μatm). Adult pairs were housed in one high‐CO_2_ system. Clutches of offspring were divided and maintained in both the control and the other high‐CO_2_ system. pH controllers (Aqua Medic, Germany) maintained the desired pH in the header tanks that supplied the tanks in each system (Table 1). Daily pH_NBS_ (National Bureau of Standards) and temperature measurements were taken in each tank using a pH electrode (SevenGo Pro, Mettler Toledo, Switzerland) and temperature probe (Cormark C26, Norfolk, UK). A portable CO_2_ equilibrator and infrared sensor (GMP343, Vaisala, Helsinki, Finland) verified seawater *p*CO_2_ (Hari et al., 2008; Munday, Watson, Chung, Marshall, & Nilsson, 2014). Water samples were taken for the duration of the experiment and used to determine total alkalinity by Gran titration, within 1% of certified reference material (Prof. A. Dickson, Scripps Institution of Oceanography). Salinity was obtained daily from moorings around Lizard Island that are part of Australia's Integrated Marine Observing System (IMOS). Carbonate chemistry parameters were calculated using CO2SYS (Pierrot, Lewis, & Wallace, 2006) with the constants K1 from Mehrbach, Culberson, Hawley, and Pytkowicz (1973) refit by Dickson and Millero (1987), and Dickson (1990) for KHSO_4_‐ (Table 1).

**Table 1:** Mean (±*SD*) seawater parameters in the experimental system for adults and juveniles during the experimental seasons

Water system	pH_NBS_	Temperature (°C)	Salinity	TA (μmol kg^−1^ SW)	*p*CO_2_ (μatm)
Field					
Control	8.13 (±0.03)	27.3 (±0.5)	35.4 (±0.02)	2,267 (±30)	452 (±37)
High CO_2_	7.95 (±0.01)	27.5 (±0.5)	35.4 (±0.02)	2,281 (±37)	754 (±23)
Laboratory					
Control	8.15 (±0.04)	28.5 (±0.2)	35.0 (±1.2)	2,146 (±125)	414 (±46)
High CO_2_	7.94 (±0.04)	28.5 (±0.3)	35.1 (±1.2)	2,223 (±152)	754 (±92)

Temperature, pH, salinity and total alkalinity (TA) were measured directly. *p*CO_2_ was estimated from these parameters using CO2SYS. Seawater parameters were consistent for breeding and experimental components of the study.

#### 2.4.2 Laboratory‐reared population

Two 10,000‐L recirculating aquarium systems were set to a different pH and corresponding CO_2_ level: a current‐day control (414 μatm) and a mid‐level end‐of‐century CO_2_ (754 μatm). CO_2_ was dosed into a 3,000‐L sump using an Aqua Medic AT Control System (Aqua Medic, Germany). This allowed for maintenance of the desired pH level in each system. The equilibrated seawater was then delivered to the holding aquaria at a rate of 1.5 L/min. Daily pH_NBS_ and temperature measurements were taken using a pH electrode (SevenGo Pro, Mettler Toledo, Switzerland) and temperature probe (Cormark C26, Norfolk, UK). Weekly salinity readings were measured using a conductivity sensor (HQ15d; Hach, Loveland, CO, USA). Total alkalinity was estimated weekly using Gran titration (Metrohm 888 Titrando Titrator Metrohm AG, Switzerland) and using certified reference material from Dr. A.G. Dickson (Scripps Institution of Oceanography). Carbonate chemistry parameters were calculated using CO2SYS as described above (Table 1).

### 2.5 Response to CAC

Response to CAC was tested in a two‐channel choice flume. The size of the chamber differed between adults (30 cm × 13 cm) (Heuer, Welch, Rummer, Munday, & Grosell, 2016) and juveniles (13 cm × 4 cm) (Gerlach, Atema, Kingsford, Black, & Miller‐Sims, 2007). Individuals were given the choice between two water streams in the flume: seawater containing conspecific CAC versus untreated seawater. Water chemistry in the flume matched the respective treatment for each fish. Water from the two different sources was gravity fed into the choice flume, which is divided down half of its length. A constant flow rate of 450 and 100 ml/min was maintained for the adults and juveniles, respectively. Flow rates were monitored using a flow meter and dye test after every water change.

To produce CAC, control donor fish were euthanized with a quick blow to the head. Donor fish were the same size and approximate age as the fish being flumed in each trial. Superficial cuts were made along the sides of the donor fish. Adult donors were then rinsed with 60 ml of control water, while juvenile donors were rinsed with 15 ml of control water (Ferrari, Dixson, et al., 2011). The rinse water was collected and immediately mixed with 10 L of treatment water in the tank used to supply CAC to the flume. CAC was replenished after every second fish to ensure a consistent concentration of fresh CAC for the duration of each trial. A ratio of one donor fish to one test fish was used.

For each trial, a single test fish was placed in the centre of a downstream end of the choice flume and given a 2‐min acclimation period. The position of the fish was then recorded every 5 s for a total of 2 min. A rest period of 1 min followed, during which time the water sources were switched and the fish was re‐centred in the downstream end of the flume. The entire acclimation and trial process was then repeated to eliminate potential side preference.

#### 2.5.1 Behaviour repeatability

A total of 20 adults were tested for repeatability of olfactory behaviour: 10 individuals from the field‐caught population and 10 individuals from the laboratory‐reared population. An equal number of males and females were tested. Individual olfactory behaviour was measured in the two‐channel choice flume, following the protocol above. Individuals were placed into separate holding tanks for 24 hr after the first behavioural trial. The same olfactory behaviour test was then repeated. Per cent time spent in the chemical alarm cue was correlated for the first and second trials to estimate trait repeatability.

### 2.6 Data analysis

The average time in CAC for each group of offspring (mid‐offspring value) was used in the parent–offspring regressions (Åkesson, Bensch, Hasselquist, Tarka, & Hansson, 2008). We focused our analysis on the father–mid‐offspring regression as this should provide the least biased estimate of narrow‐sense heritability (Falconer & Mackay, 1996). Heritability (*h* ^2^) was calculated as two times the slope of the least‐squares regression between the father and mid‐offspring values (Lynch & Walsh, 1998). Any negative estimates of heritability were interpreted as zero.

ANCOVA was then used to compare regressions among the different treatment groups, with offspring treatment as the categorical factor and father olfactory response as the covariate. This analysis allowed us to test whether (i) heritability (the slope of the regression) differed between treatments groups and (ii) whether the magnitude of the behavioural response to CAC (intercept) differed between juvenile fish reared in control versus high CO_2_, between acute versus chronic high‐CO_2_ treatments and between offspring from control versus high‐CO_2_ parents. A homogeneity of slopes model was used to test for an interaction between the main effects and the covariate, which would indicate that *h* ^2^ differed among treatment groups. Where no significant interaction was detected, the model was rerun with the interaction term removed to test for differences in elevation between treatment groups.

## 3 RESULTS

### 3.1 Field‐collected population

**Figure 2:**
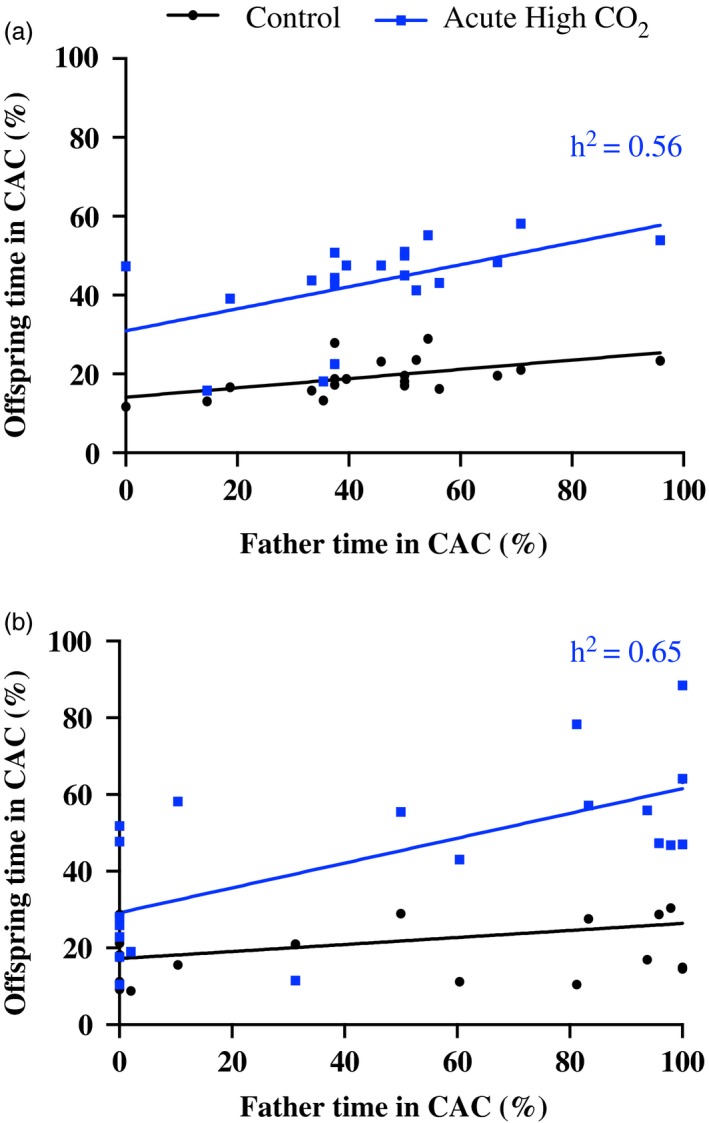
Father–mid‐offspring regressions for olfactory preference of fish acutely exposed to high CO_2_ for (a) the field‐collected population and (b) the laboratory‐reared population. Per cent time spent in the chemical alarm cue was measured. Father responses are from acute CO_2_ exposure and are plotted along the *x*‐axes. Mid‐offspring responses are plotted along the *y*‐axes for direct relationships with their fathers. Regressions are grouped by offspring treatments: control and acute high CO_2_. Laboratory‐reared juveniles are from parents held in ambient control during the breeding season. *h* ^2^ = 2*b* and is depicted on the graphs

Acute exposure to elevated CO_2_ altered juvenile olfactory response to CAC, with two to four times more time spent in CAC for CO_2_‐treated fish compared to sibling fish kept in current‐day control seawater (Figure 2a; *F*_1,36_ = 7.97, *p *<* *.01).

Heritability in the behavioural response to CAC was high (0.56) in wild‐caught fish acutely exposed to high CO_2_ (Table 2) indicating a large component of additive genetic variation. This estimate was strongly influenced by offspring from three of the 20 fathers. Offspring from these three fathers exhibited a response to CAC that was more similar to control fish (i.e., low time in CAC) compared with other offspring acutely exposed to high CO_2_ (Figure 2a). The average time in CAC for these high‐CO_2_ offspring was only 2%–5% more than their siblings that had been reared and tested in control conditions. Heritability declines to 0.26 (±0.11 *SE*) if these three families with tolerant offspring responses are removed from the analysis.

**Table 2:** Heritability (*h* ^2^) of olfactory behaviour for offspring in elevated CO_2_ conditions estimated from father–mid‐offspring regressions

Parent holding condition	Juvenile treatment	Population	*h* ^2^ = 2*b*	*SE* a	*N*
Control	Acute high CO_2_	Field‐collected	0.56	0.23	20
	Laboratory‐reared	0.65	0.17	20
		Chronic high CO_2_	0.02	0.11	20
High CO_2_		Acute high CO_2_	0.28	0.09	18
		Chronic high CO_2_	−0.01	0.06	18

Negative heritabilities are displayed for reference, but should be interpreted as 0.00. Heritabilities (to the nearest 0.01), standard error (*SE*) and sample size (*N*) are shown.

a *SE* of *h* ^2^ is twice the standard error of the slope *b*.

Individual repeatability in responses to CAC was very high for field‐caught fish with a correlation of *r* = .992 (*p *<* *.001) between the first and second trial for time spent in CAC by adults exposed to high CO_2_.

### 3.2 Laboratory‐reared population

Similar to the field‐caught population, juveniles that were acutely exposed to high CO_2_ spent two to seven times more time in CAC than sibling fish kept in current‐day control seawater (Figure 2b; *F*_1,36_ = 3.23, *p *>* *.05).

Heritability in the behavioural response to CAC was high (0.65) in the laboratory‐reared fish that were acutely exposed to high CO_2_ (Table 2) indicating a large additive genetic component. Heritability estimated in the laboratory‐reared fish that were acutely exposed to high CO_2_ (±0.17 *SE*) overlapped with the estimate of heritability in the wild‐caught fish acutely exposed to high CO_2_ (0.56 ± 0.23 *SE*).

In direct contrast to the acutely exposed fish, heritability was very low (0.02) for siblings that had been chronically exposed to high CO_2_ from hatching (Table 2). With a *SE* of ±0.11, the estimate of heritability for fish that were chronically exposed to high CO_2_ could not be distinguished from zero.

**Figure 3:**
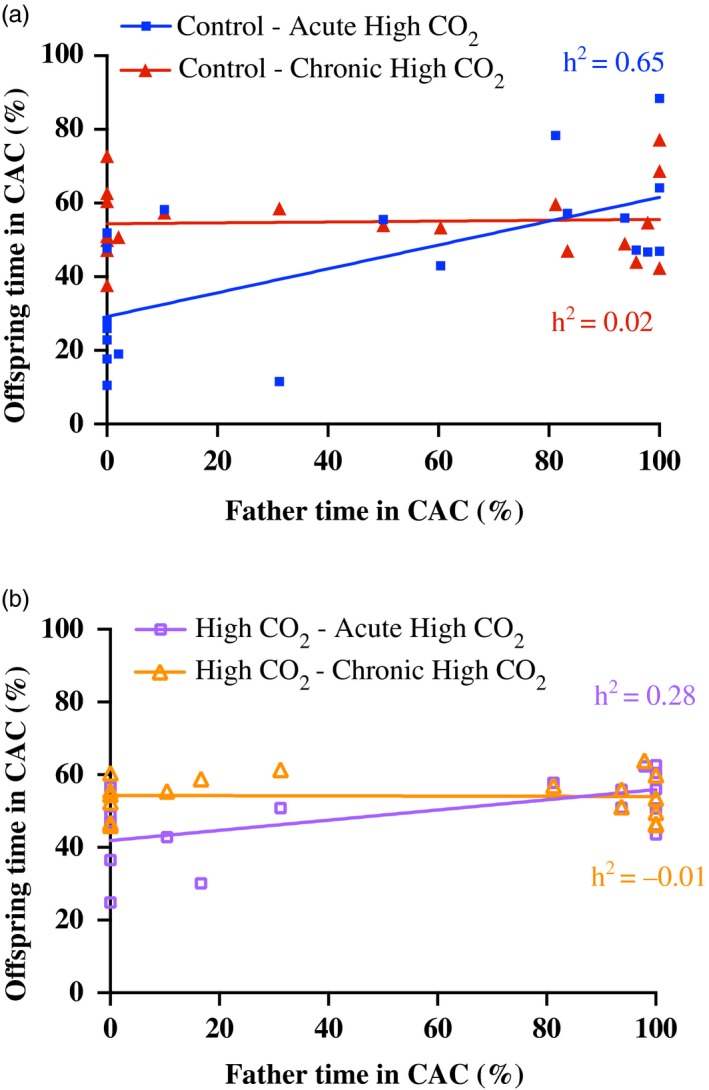
Acute high‐CO_2_ versus chronic high‐CO_2_ effects on olfactory responses for father–mid‐offspring regressions in the laboratory‐reared population. Per cent time spent in the chemical alarm cue was measured. Father responses are from acute CO_2_ exposure and are plotted along the *x*‐axes. Mid‐offspring responses are plotted along the *y*‐axes for direct relationships with their fathers. Regressions are grouped by offspring treatments, with the first word in the legend indicating their fathers’ holding condition (a) control (414 μatm) or (b) high CO_2_ (754 μatm), and the second word in the legend indicating offspring rearing treatment (Acute high CO_2_ or Chronic high CO_2_). *h* ^2^ = 2*b* and is depicted on the graphs

The comparison of regression slopes (Figure 3) further demonstrates the effects of acute versus chronic high‐CO_2_ exposure on heritability of behavioural response to CAC. For offspring from control fathers, there was a significant interaction between the slopes for acute high‐CO_2_ offspring and chronic high‐CO_2_ offspring (Figure 3a; *F*_1,36_ = 9.55, *p *<* *.01). This interaction was due to the positive relationship between fathers and their offspring that were acutely treated with high CO_2_, compared with no relationship for the same fathers and sibling offspring that were chronically exposed to high CO_2_.

Heritability in the behavioural response to CAC for fish from parents exposed to high CO_2_ for 3 months before breeding was moderate (0.28) in offspring acutely exposed to high CO_2_ and could not be distinguished from zero for offspring chronically exposed to high CO_2_ (Table 2). For fish that were acutely exposed to high CO_2_, heritability was lower in the group from parents maintained continuously in high CO_2_ (0.28) compared with the group from parents that were maintained in control conditions after initial behavioural testing (0.65), indicating a negative effect of parental CO_2_ exposure on heritability in the behavioural response to CAC.

Comparison of regression slopes showed a significant interaction between the acute high‐CO_2_ offspring and chronic high‐CO_2_ offspring from high‐CO_2_ fathers (holding condition) (Figure 3b; *F*_1,32_ = 7.29, *p *<* *.05). This interaction was similar to that seen between offspring from control fathers, resulting from the positive relationship between fathers and their offspring that were acutely treated with high CO_2_, but lack of relationship for the same fathers and sibling offspring that were chronically exposed to high CO_2_. Indeed, when compared with the same offspring treatments (Figure 3a,b) from parents held in control conditions, there was an overwhelming effect of acute versus chronic high‐CO_2_ exposure on heritability and a relatively minimal effect of parental CO_2_ treatment on heritability.

As observed in field‐collected fish, repeatability was very high for laboratory‐reared fish with a correlation of *r* = .997, *p *<* *.001, between the first and second trial for adults exposed to high CO_2_.

## 4 DISCUSSION

We found that offspring exposed to elevated CO_2_ exhibited impaired antipredator behaviour, spending an increased amount of time in a water stream containing CAC compared with control fish, consistent with previous findings (e.g., Ferrari, Dixson, et al., 2011; Welch et al., 2014). More importantly, our father–offspring regressions revealed strong heritability of variation in behavioural response to CAC in offspring that are acutely exposed to high CO_2_, but there was no heritability in this trait when offspring were chronically exposed to high CO_2_. Our results show that parents that were tolerant to acute elevated CO_2_ conditions produced offspring that also exhibited behavioural tolerance under acute high‐CO_2_ treatments, but this effect was not evident in sibling offspring that were reared in high CO_2_ from hatching. Parental exposure to high CO_2_ also diminished the heritability of behavioural tolerance to high CO_2_, but not to the same extent as chronic exposure to high CO_2_ in juveniles. These results have important ramifications for understanding the likelihood that fish will be able to adapt to projected future CO_2_ levels in the ocean.

Our results show that the duration of exposure to high CO_2_ can substantially influence the variation in behavioural responses exhibited in juvenile fish, and thus the heritability of behavioural responses to high CO_2_. In the laboratory‐reared population, there was high heritability of behavioural response to CAC in offspring that had been acutely exposed to high CO_2_, but not in offspring that had been chronically exposed to high CO_2_. Importantly, fish in these two treatments were siblings and there was almost no mortality during the 6‐week rearing period that could have led to the selection of less tolerant genotypes; therefore, the difference must be due to plasticity, not genetic differences. The reduction in heritability for offspring chronically exposed to high CO_2_ occurred due to previously tolerant offspring (low percentage time in CAC) in acute high CO_2_ losing this tolerance in the chronically exposed siblings (high percentage time in CAC). When chronically exposed to high CO_2_, all juveniles exhibited a high percentage time in CAC. This suggests that the behavioural phenotype of CO_2_‐tolerant offspring is modified by nonadaptive plasticity when they are chronically exposed to high CO_2_. Innate responses in animals, such as predator and CAC avoidance, tend to occur rapidly and be both favourable to the individual and heritable (Agrawal, Laforsch, & Tollrian, 1999). However, individual variation in responses may diminish in the longer term due to the convergence of cellular and physiological processes in a common environment (Day & Bonduriansky, 2011). Here, we observed a reduction in phenotypic variation expressed in juveniles that were chronically exposed to high CO_2_ compared with juveniles that were acutely exposed to high CO_2_, causing a reduction in the heritability of behavioural response to CAC in a high‐CO_2_ environment. While parent behaviours were only recorded after acute high‐CO_2_ exposure for this experiment, it is likely that heritability would remain low for chronically exposed juveniles if parent behaviours were measured after chronic high‐CO_2_ exposure due to the reduction in phenotypic variation seen in our results. Our results are consistent with reduced heritability in unfavourable conditions in other animals (Charmantier & Garant, 2005; Wilson et al., 2006), but they also demonstrate that the mechanism may be nonadaptive plasticity in stressful environments. Critically, our results suggest that the collapse in phenotypic variation under chronically high‐CO_2_ conditions may reduce the potential for adaptation of fish populations to consistently high‐CO_2_ levels in the future.

High heritabilities for behavioural response to CAC in the acute high‐CO_2_ treatment in both the field‐caught and laboratory‐reared populations were strongly driven by a few families that exhibited a high behavioural tolerance to high CO_2_. Offspring from these families had similar phenotypes in both high CO_2_ and control conditions, spending minimal time in CAC, indicating that their behavioural response to CAC was tolerant to the effects of high CO_2_. This is consistent with previous observations that some individuals appear to be completely unaffected by this level of CO_2_ (700 μatm) and exhibit no change in behaviour compared with controls (Munday et al., 2010). Furthermore, these highly tolerant individuals are selectively favoured in their natural habitat because they suffer lower rates of predation (Munday et al., 2012). These earlier observations suggested that there could be rapid adaption of behavioural tolerance to high CO_2_ in fish populations. However, those studies involved juveniles that had been acutely exposed to high CO_2_ for the same duration as our acute treatments. Our results indicate that these individuals may lack this behavioural tolerance when chronically exposed to high CO_2_ from hatching, and this will constrain the adaptive potential of fish populations to high CO_2_.

There was a negative effect of parent holding conditions on the heritability of behavioural response to CAC in fish that were acutely exposed to high CO_2_, with a reduction in *h* ^2^ from 0.65 to 0.28 for fathers maintained in control versus high‐CO_2_ conditions, respectively. This suggests that nongenetic parental effects might reduce variation in the behavioural response to high CO_2_ and consequently reduce the pace of adaptation. Heritable variation was present in offspring treated with acute high CO_2_, indicating that adaptation would be expected to occur in instances where individuals experience short‐term exposure to high‐CO_2_ levels, as might occur in natural upwelling zones and coastal habitats (Hofmann et al., 2011). Nevertheless, higher anthropogenic CO_2_ levels will be permanent in the future, exposing successive generations of individuals to high CO_2_ for extensive periods of time, which might reduce adaptive potential. Our chronic CO_2_ treatment sought to mirror this scenario, and there was no additive genetic variation observed, regardless of parental treatment. Similar to Welch et al. (2014), our results also showed no evidence for transgenerational acclimation of behavioural response to high CO_2_ because there was an increased attraction to CAC compared with controls for all offspring that were chronically exposed to high CO_2_, regardless of the parental CO_2_ holding condition.

There was heritable phenotypic variation in behavioural responses of juvenile fish exposed to high CO_2_ for 4 days, but this variation was absent in fish reared for 6 weeks in high CO_2_. However, the rate of decay in phenotypic variation through time is unknown. The rate of decay could be important because mortality rates of reef fish from predation are highest in small size classes and diminish rapidly with increasing size and age (Almany & Webster, 2006; Jones & McCormick, 2002). Therefore, the strongest selection for appropriate behavioural responses to the threat of predation is likely to occur at an early age when juveniles will have had a shorter period of time exposed to high CO_2_. It is possible that selection of CO_2_‐tolerant phenotypes could occur during this critical window of early life, leading to the evolution of more CO_2_‐tolerant populations, even if that tolerance is obscured by phenotypic plasticity later in life. Further studies assessing the rate of decay in phenotypic variation through time are required to determine the window of opportunity for selection to act on genetic variation in the behavioural response to predation threat in a high CO_2_ environment.

We used parent–offspring regressions to estimate heritability of variation in the behavioural response of fish to CAC in a high‐CO_2_ environment. Assortative mating can influence estimates of heritability in parent–offspring regressions; in particular, it may generate higher estimates of heritability than random mating (Lynch & Walsh, 1998). We assumed that natural breeding pairs in the field were mated randomly, and thus, our estimates of heritability from the field population are unlikely to be biased by assortative mating. However, our laboratory breeding pairs were deliberately constructed to include pairs of similar high or low behavioural tolerance to high CO_2_. This assortative mating design had the potential to inflate our estimate of heritability. Nevertheless, there was no significant difference in heritability estimated between the field‐caught and laboratory‐reared populations for the acute high‐CO_2_ treatments, with both populations demonstrating high heritabilities of behavioural response to CAC in the acute high‐CO_2_ treatment. Our estimate of heritability in acutely exposed fish from the laboratory population (0.65) was higher than from the field‐caught population (0.56), but the values were not significantly different due to the relatively large standard errors (±0.23). It is possible that with a larger sample size, these values might have been significantly different. More critical to our findings, however, is that the assortative mating design would have produced the highest estimate of heritability in the fish that were chronically exposed to high CO_2_. Despite a possible positive bias, our estimate of heritability in the chronic CO_2_ treatment was extremely low with an error range that encompassed zero. Consequently, we have increased confidence that there is negligible heritability of behavioural response to CAC when juvenile fish have been permanently exposed to high CO_2_ from hatching.

While father–mid‐offspring regressions provide the best possible estimate of narrow‐sense heritability, they may still include multiple sources of genetic variation (Lynch & Walsh, 1998). There may also be nongenetic effects, such as from parental egg care (Robertson, 1973), or other environmental factors that could affect phenotypic variation in parents and their offspring. If our estimates of heritability from father–mid‐offspring regressions contained a large environmental component, we would expect to see very different heritability values between the field‐caught and laboratory‐reared populations due to substantial differences in the general environments they experienced and because the offspring were separated from their parents at hatching in the laboratory‐reared population, but had ongoing parental care in the wild‐caught population. Despite the many environmental differences that adults and their offspring would have experienced in the two populations, the estimated heritability of behavioural response to CAC in the field‐caught and laboratory‐reared populations was remarkably similar. This similarity in heritability estimates, despite the substantial environmental differences between populations, supports the presence of a large genetic component to the phenotypic variation in behavioural response to high CO_2_ in acutely exposed fish. In another recent study, Schunter et al. (2016) found that patterns of gene expression in the brain of juvenile *A. polyacanthus* exhibited a strong connection to the assignment of their parents as either tolerant or nontolerant to high CO_2_, which is also consistent with the presence of genetic variation in CO_2_ tolerance for this species.

Our results show that heritability in behavioural responses to high CO_2_ is obscured by nonadaptive phenotypic plasticity when juveniles are exposed to high CO_2_ for prolonged periods of time. Reef fishes initially display heritable phenotypic variation in behavioural response to high CO_2_ that could favour selection of tolerant genotypes during a short ontogenetic window, but this phenotypic variation is lost with longer exposure to high CO_2_. This underscores the importance of investigating genetic variation in phenotypic traits over time scales relevant to environmental change in natural habitats. More generally, our results demonstrate the potential difficulty in estimating adaptive potential to a rapidly changing environment because estimates of heritability of phenotypic variation can differ markedly depending on the duration of the environmental stress applied in experiments. Our results also highlight the potential importance of nonadaptive plasticity to estimating evolutionary potential. Theory and empirical research has generally concentrated on whether adaptive plasticity constrains or facilitates adaptive evolution (Hendry, 2015; Merilä, 2015), with little consideration of nonadaptive plasticity. However, in a recent study, Ghalambor et al. (2015) showed that nonadaptive plasticity might promote adaptive evolution by increasing the strength of natural selection. In contrast, our results suggest that nonadaptive plasticity could constrain adaptive evolution by reducing the phenotypic variation, and thus heritability, of traits expressed in some environments. Either way, considering the role of plasticity in facilitating or constraining adaptive potential will be critical to making reliable predictions about the future of animal populations under rapid climate change.

## CONFLICT OF INTEREST

The authors declare no conflict of interests or competing financial interests.

## DATA ARCHIVING

Data for this study are available at https://doi.org/10.5061/dryad.dc068.
